# Dimethyl­ammonium tetra­chloridoferrate(III) 18-crown-6 clathrate

**DOI:** 10.1107/S1600536810020192

**Published:** 2010-06-05

**Authors:** Ping Ping Shi, Min Min Zhao

**Affiliations:** a Ordered Matter Science Research Center, College of Chemistry and Chemical Engineering, Southeast University, Nanjing 211189, People’s Republic of China

## Abstract

The reaction of dimethyl­amine hydro­chloride, 18-crown-6 and ferric chloride in ethanol yields the title compound, (C_2_H_8_N)[FeCl_4_]·C_12_H_24_O_6_, which exhibits an unusual supramolecular structure. The protonated dimethyl­amine contains one NH_2_^+^ group, resulting in a 1:1 supra­molecular rotator–stator structure (CH_3_—NH_2_^+^—CH_3_)(18-crown-6), through N—H⋯O hydrogen-bonding inter­actions between the ammonium group of the cation and the O atoms of the crown ether. In the crystal, all three components lie on a common crystallographic mirror plane normal to [010].

## Related literature

For similar 18-crown-6 clathrates, see: Akutagawa *et al.* (2002 ▶); Fender *et al.* (2002 ▶). For the ferroelectric properties of these materials, see: Zhang *et al.* (2009 ▶); Ye *et al.* (2009 ▶).
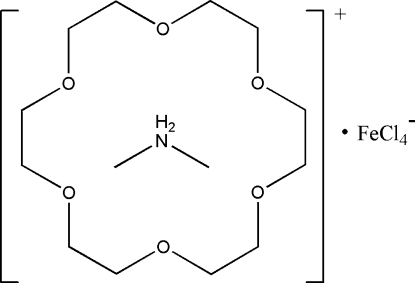


## Experimental

### 

#### Crystal data

(C_2_H_8_N)[FeCl_4_]·C_12_H_24_O_6_
*M* *_r_* = 508.06
Orthorhombic, 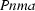

*a* = 9.3035 (19) Å
*b* = 11.328 (2) Å
*c* = 23.230 (5) Å
*V* = 2448.1 (9) Å^3^
*Z* = 4
Mo *K*α radiation
μ = 1.08 mm^−1^
*T* = 293 K
0.40 × 0.30 × 0.20 mm

#### Data collection

Rigaku SCXmini diffractometer
Absorption correction: multi-scan (*CrystalClear*; Rigaku, 2005 ▶) *T*_min_ = 0.685, *T*_max_ = 0.806
23771 measured reflections
2940 independent reflections
1799 reflections with *I* > 2σ(*I*)
*R*_int_ = 0.073

#### Refinement

*R*[*F* ^2^ > 2σ(*F* ^2^)] = 0.052
*wR*(*F* ^2^) = 0.128
*S* = 0.99
2940 reflections
130 parameters
H-atom parameters constrained
Δρ_max_ = 0.33 e Å^−3^
Δρ_min_ = −0.23 e Å^−3^

### 

Data collection: *CrystalClear* (Rigaku, 2005 ▶); cell refinement: *CrystalClear*; data reduction: *CrystalClear*; program(s) used to solve structure: *SHELXS97* (Sheldrick, 2008 ▶); program(s) used to refine structure: *SHELXL97* (Sheldrick, 2008 ▶); molecular graphics: *SHELXTL* (Sheldrick, 2008 ▶); software used to prepare material for publication: *PRPKAPPA* (Ferguson, 1999 ▶).

## Supplementary Material

Crystal structure: contains datablocks I, global. DOI: 10.1107/S1600536810020192/bh2286sup1.cif

Structure factors: contains datablocks I. DOI: 10.1107/S1600536810020192/bh2286Isup2.hkl

Additional supplementary materials:  crystallographic information; 3D view; checkCIF report

## Figures and Tables

**Table 1:** Hydrogen-bond geometry (Å, °)

*D*—H⋯*A*	*D*—H	H⋯*A*	*D*⋯*A*	*D*—H⋯*A*
N1—H1*C*⋯O2^i^	0.90	2.03	2.867 (3)	155

Symmetry code: (i) 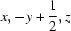.

**Fig. 1.**
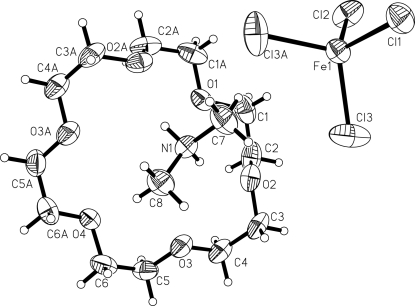
The title compound, with the atomic numbering scheme. The displacement ellipsoids are drawn at the 30% probability level.

**Fig. 2.**
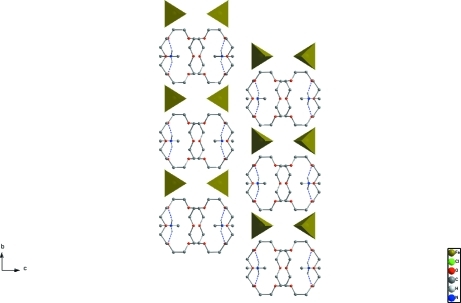
A view of the structure along the a axis. The dashed lines depict the hydrogen bonds.

## supplementary crystallographic information

### Comment

There is currently a great deal of interest in crown ethers because of their ability to form non-covalent, H-bonding complexes with ammonium cations, both in solid and in solution (Akutagawa *et al.*, 2002; Fender *et al.*, 2002). Not only the size of the crown ether, but also the nature of the ammonium cation (NH_4_^+^, *R*NH_3_^+^, *R*_2_NH_2_^+^, *etc.*) can influence on the stoichiometry and stability of these host–guest complexes. The host molecules combine with the guest species by intermolecular interactions, and, if the host molecule contains some specific sites, it is easy to realise high selectivity in ion or molecular recognition. 18-Crown-6 has the highest affinity for ammonium cations *R*NH_3_^+^. While most studies of 18-crown-6 and its derivatives invariably showed a 1:1 stoichiometry with *R*NH_3_^+^ cations, some structurally characterized complexes of crown ethers include *R*_2_NH_2_^+^ cations.

The present study is a part of systematic investigation of ferroelectric, phase transitions materials (Ye *et al.*, 2009; Zhang *et al.*, 2009) that include metal-organic coordination compounds with organic ligands, or are related to the structures with both organic and inorganic building fragments. In the measured temperature range from 80 to 420 K (m.p. > 430 K), the temperature dependence of the relative permittivity at 1 MHz varied smoothly from 4.6 to 7.2 in the title compound. No dielectric anomaly has been observed. This suggests that this compound is not an actual ferroelectric, or that no distinct phase transition occurred within the probed temperature range.

The title compound is composed of cationic [C_2_H_8_N (18-Crown-6)]^+^ and one single anionic [FeCl_4_]^-^ complex (Fig. 1). Supramolecular rotators are assembled between protonated dimethylamine (CH_3_—NH_2_—CH_3_)^+^ and 18-crown-6 by hydrogen-bonding. The ammonium moieties of (NH_2_^+^) cations interact with two O atoms of the crown ether through two simple N—H···O hydrogen bonds, forming a 1:1 supramolecular rotator-stator structure.

Supramolecular cation structure, [C_2_H_8_N (18-Crown-6)]^+^, were introduced as counter cations to [FeCl_4_]^-^ anions. The crown adopts a conformation in which the ring shows some distortion from the mean plane, with the torsion angles: C3—O2—C2—C1 = 178.2 (3)°; C2—O2—C3—C4 = 72.6 (4)°; C5—O3—C4—C3=178.9 (3)°; C4—O3—C5—C6 = 179.5 (3)°; O3—C4—C3—O2=61.0 (5)°. Fe(III) has a flattened tetrahedral coordination by four Cl^-^ ions [range of *cis*-bond angles: 108.14 (4)–111.67 (9)°; dav (Fe—Cl) = 2.1728 (15)–2.1889 (12) Å].

Fig. 2 shows a view of the crystal structure down the *a* axis. An alternate arrangement of cations and anions layers is elongated along the *b* axis. The title compound is stabilized by intramolecular N—H···O hydrogen bonds, but no significant intermolecular hydrogen bonds are observed.

### Experimental

CH_3_—NH—CH_3_.HCl (2 mmol, 0.163 g) and 18-crown-6 (2 mmol, 0.528 g) were dissolved in ethanol. Then, trivalent ferric chloride (2 mmol, 0.54 g) was added to the mixture in concentrated hydrochloric acid medium, the precipitate was filtered and washed with a small amount of ethanol. Five days later, single crystals suitable for X-ray diffraction analysis were obtained from slow evaporation of ethanol and DMF solution at room temperature.

### Refinement

All C-bonded H atoms were calculated geometrically with C—H distances fixed to 0.96 Å, and were allowed to ride on the C atoms to which they are bonded, with *U*_iso_(H) = 1.2*U*_eq_(C) or *U*_iso_(H) = 1.5*U*_eq_(C) (methyl groups). The ammonium H atom (H1C) was calculated geometrically and refined using a riding model with N—H = 0.90 Å and *U*_iso_(H) = 1.2*U*_eq_(N).

### Crystal data

**Table:**

(C_2_H_8_N)[FeCl_4_]·C_12_H_24_O_6_	*F*(000) = 1060
*M**_r_* = 508.06	*D*_x_ = 1.378 Mg m^−^^3^
Orthorhombic, *P**n**m**a*	Mo *K*α radiation, λ = 0.71073 Å
Hall symbol: -P 2ac 2n	Cell parameters from 17071 reflections
*a* = 9.3035 (19) Å	θ = 3.2–27.8°
*b* = 11.328 (2) Å	µ = 1.08 mm^−^^1^
*c* = 23.230 (5) Å	*T* = 293 K
*V* = 2448.1 (9) Å^3^	Prism, yellow
*Z* = 4	0.40 × 0.30 × 0.20 mm

### Data collection

**Table:**

Rigaku SCXmini diffractometer	2940 independent reflections
Radiation source: fine-focus sealed tube	1799 reflections with *I* > 2σ(*I*)
graphite	*R*_int_ = 0.073
Detector resolution: 13.6612 pixels mm^-1^	θ_max_ = 27.5°, θ_min_ = 3.2°
CCD_Profile_fitting scans	*h* = −12→12
Absorption correction: multi-scan (*CrystalClear*; Rigaku, 2005)	*k* = −14→14
*T*_min_ = 0.685, *T*_max_ = 0.806	*l* = −30→30
23771 measured reflections	

### Refinement

**Table:**

Refinement on *F*^2^	Primary atom site location: structure-invariant direct methods
Least-squares matrix: full	Secondary atom site location: difference Fourier map
*R*[*F*^2^ > 2σ(*F*^2^)] = 0.052	Hydrogen site location: inferred from neighbouring sites
*wR*(*F*^2^) = 0.128	H-atom parameters constrained
*S* = 0.99	*w* = 1/[σ^2^(*F*_o_^2^) + (0.04*P*)^2^ + 1.99*P*] where *P* = (*F*_o_^2^ + 2*F*_c_^2^)/3
2940 reflections	(Δ/σ)_max_ < 0.001
130 parameters	Δρ_max_ = 0.33 e Å^−^^3^
0 restraints	Δρ_min_ = −0.23 e Å^−^^3^
0 constraints	

### Fractional atomic coordinates and isotropic or equivalent isotropic displacement parameters (Å^2^)

**Table:**

	*x*	*y*	*z*	*U*_iso_*/*U*_eq_	
Fe1	0.47623 (7)	0.2500	0.38202 (3)	0.0633 (2)	
Cl2	0.25400 (14)	0.2500	0.35240 (7)	0.0926 (5)	
Cl1	0.61871 (16)	0.2500	0.30791 (6)	0.0890 (4)	
Cl3	0.51101 (12)	0.09011 (13)	0.43307 (5)	0.1218 (5)	
O1	−0.1251 (4)	0.2500	0.57334 (13)	0.0776 (10)	
O2	0.0327 (3)	0.0373 (2)	0.58570 (10)	0.0804 (7)	
O4	0.0916 (4)	0.2500	0.76754 (13)	0.0731 (9)	
O3	0.1171 (3)	0.0390 (2)	0.70458 (11)	0.0814 (7)	
C2	−0.1174 (4)	0.0408 (4)	0.5777 (2)	0.0991 (14)	
H2A	−0.1646	0.0442	0.6144	0.119*	
H2B	−0.1483	−0.0298	0.5584	0.119*	
C4	0.0511 (5)	−0.0599 (3)	0.67999 (18)	0.0899 (12)	
H4A	−0.0510	−0.0556	0.6856	0.108*	
H4B	0.0856	−0.1305	0.6982	0.108*	
C6	0.1585 (5)	0.1474 (4)	0.78905 (16)	0.0929 (12)	
H6A	0.1503	0.1454	0.8302	0.111*	
H6B	0.2587	0.1480	0.7793	0.111*	
C3	0.0837 (5)	−0.0633 (3)	0.61760 (18)	0.0901 (12)	
H3A	0.1859	−0.0689	0.6128	0.108*	
H3B	0.0418	−0.1334	0.6014	0.108*	
C1	−0.1581 (5)	0.1448 (4)	0.54302 (18)	0.1033 (15)	
H1A	−0.1071	0.1438	0.5071	0.124*	
H1B	−0.2592	0.1424	0.5348	0.124*	
C5	0.0908 (5)	0.0448 (4)	0.76473 (16)	0.0910 (12)	
H5A	0.1280	−0.0249	0.7830	0.109*	
H5B	−0.0108	0.0478	0.7717	0.109*	
N1	0.1943 (4)	0.2500	0.60266 (16)	0.0631 (10)	
H1C	0.1395	0.3142	0.6088	0.076*	
C8	0.3114 (6)	0.2500	0.6442 (2)	0.0885 (16)	
H8A	0.2746	0.2500	0.6828	0.133*	
H8B	0.3692	0.1808	0.6383	0.133*	
C7	0.2423 (7)	0.2500	0.5428 (2)	0.107 (2)	
H7A	0.1606	0.2500	0.5175	0.161*	
H7B	0.2992	0.3192	0.5358	0.161*	

### Atomic displacement parameters (Å^2^)

**Table:**

	*U*^11^	*U*^22^	*U*^33^	*U*^12^	*U*^13^	*U*^23^
Fe1	0.0520 (4)	0.0775 (5)	0.0602 (4)	0.000	−0.0032 (3)	0.000
Cl2	0.0602 (7)	0.0757 (9)	0.1419 (13)	0.000	−0.0276 (8)	0.000
Cl1	0.0893 (10)	0.1058 (11)	0.0719 (8)	0.000	0.0172 (7)	0.000
Cl3	0.0934 (8)	0.1508 (12)	0.1212 (9)	0.0227 (7)	0.0078 (6)	0.0725 (9)
O1	0.076 (2)	0.098 (3)	0.0589 (19)	0.000	−0.0134 (17)	0.000
O2	0.0754 (16)	0.0766 (17)	0.0892 (17)	−0.0121 (13)	0.0188 (13)	−0.0222 (14)
O4	0.080 (2)	0.076 (2)	0.064 (2)	0.000	−0.0125 (17)	0.000
O3	0.0913 (18)	0.0591 (15)	0.0937 (18)	0.0036 (13)	0.0070 (14)	−0.0007 (13)
C2	0.080 (3)	0.098 (3)	0.120 (4)	−0.019 (2)	0.008 (2)	−0.045 (3)
C4	0.097 (3)	0.058 (2)	0.115 (3)	0.007 (2)	0.017 (2)	−0.001 (2)
C6	0.092 (3)	0.113 (3)	0.074 (2)	0.018 (3)	−0.015 (2)	0.019 (2)
C3	0.103 (3)	0.050 (2)	0.117 (3)	0.003 (2)	0.031 (3)	−0.013 (2)
C1	0.082 (3)	0.145 (4)	0.083 (3)	−0.005 (3)	−0.020 (2)	−0.034 (3)
C5	0.106 (3)	0.082 (3)	0.085 (3)	0.012 (2)	−0.007 (2)	0.022 (2)
N1	0.050 (2)	0.060 (2)	0.080 (3)	0.000	0.0050 (19)	0.000
C8	0.066 (3)	0.114 (5)	0.086 (4)	0.000	−0.009 (3)	0.000
C7	0.113 (5)	0.141 (6)	0.069 (4)	0.000	0.010 (3)	0.000

### Geometric parameters (Å, °)

**Table:**

Fe1—Cl1	2.1728 (15)	C4—H4B	0.9599
Fe1—Cl2	2.1791 (14)	C6—C5	1.437 (5)
Fe1—Cl3^i^	2.1889 (12)	C6—H6A	0.9601
Fe1—Cl3	2.1889 (12)	C6—H6B	0.9600
O1—C1	1.418 (4)	C3—H3A	0.9600
O1—C1^i^	1.418 (4)	C3—H3B	0.9601
O2—C2	1.410 (4)	C1—H1A	0.9599
O2—C3	1.440 (4)	C1—H1B	0.9600
O4—C6^i^	1.410 (4)	C5—H5A	0.9600
O4—C6	1.410 (4)	C5—H5B	0.9600
O3—C4	1.399 (4)	N1—C8	1.455 (6)
O3—C5	1.420 (4)	N1—C7	1.461 (6)
C2—C1	1.476 (6)	N1—H1C	0.9000
C2—H2A	0.9601	C8—H8A	0.9599
C2—H2B	0.9600	C8—H8B	0.9601
C4—C3	1.481 (5)	C7—H7A	0.9600
C4—H4A	0.9600	C7—H7B	0.9600
			
Cl1—Fe1—Cl2	109.19 (7)	O2—C3—C4	114.5 (3)
Cl1—Fe1—Cl3^i^	109.82 (4)	O2—C3—H3A	108.6
Cl2—Fe1—Cl3^i^	108.14 (4)	C4—C3—H3A	108.6
Cl1—Fe1—Cl3	109.82 (4)	O2—C3—H3B	108.6
Cl2—Fe1—Cl3	108.14 (4)	C4—C3—H3B	108.7
Cl3^i^—Fe1—Cl3	111.67 (9)	H3A—C3—H3B	107.6
C1—O1—C1^i^	114.3 (4)	O1—C1—C2	110.1 (3)
C2—O2—C3	114.6 (3)	O1—C1—H1A	109.6
C6^i^—O4—C6	111.1 (4)	C2—C1—H1A	109.7
C4—O3—C5	111.3 (3)	O1—C1—H1B	109.6
O2—C2—C1	110.4 (4)	C2—C1—H1B	109.6
O2—C2—H2A	109.7	H1A—C1—H1B	108.2
C1—C2—H2A	109.5	O3—C5—C6	110.4 (3)
O2—C2—H2B	109.6	O3—C5—H5A	109.6
C1—C2—H2B	109.5	C6—C5—H5A	109.4
H2A—C2—H2B	108.1	O3—C5—H5B	109.6
O3—C4—C3	109.3 (3)	C6—C5—H5B	109.6
O3—C4—H4A	109.8	H5A—C5—H5B	108.1
C3—C4—H4A	109.7	C8—N1—C7	113.7 (4)
O3—C4—H4B	109.9	C8—N1—H1C	108.6
C3—C4—H4B	109.9	C7—N1—H1C	109.0
H4A—C4—H4B	108.3	N1—C8—H8A	110.6
O4—C6—C5	109.5 (3)	N1—C8—H8B	108.9
O4—C6—H6A	109.7	H8A—C8—H8B	109.5
C5—C6—H6A	109.8	N1—C7—H7A	109.9
O4—C6—H6B	109.8	N1—C7—H7B	109.3
C5—C6—H6B	109.8	H7A—C7—H7B	109.5
H6A—C6—H6B	108.2		

Symmetry codes: (i) *x*, −*y*+1/2, *z*.

### Hydrogen-bond geometry (Å, °)

**Table:**

*D*—H···*A*	*D*—H	H···*A*	*D*···*A*	*D*—H···*A*
N1—H1C···O2^i^	0.90	2.03	2.867 (3)	155

Symmetry codes: (i) *x*, −*y*+1/2, *z*.
